# Genetic interactions support an inhibitory relationship between bone morphogenetic protein 2 and netrin 1 during semicircular canal formation

**DOI:** 10.1242/dev.174748

**Published:** 2019-02-15

**Authors:** Chan Ho Hwang, James Keller, Charles Renner, Sho Ohta, Doris K. Wu

**Affiliations:** National Institute on Deafness and Other Communication Disorders, National Institutes of Health, Porter Neuroscience Research Center, Bethesda, MD 20892, USA

**Keywords:** Patterning, Inner ear, Morphogenesis, Mouse

## Abstract

The semicircular canals of the mammalian inner ear are derived from epithelial pouches in which epithelial cells in the central region of each pouch undergo resorption, leaving behind the region at the rim to form a tube-shaped canal. Lack of proliferation at the rim and/or over-clearing of epithelial cells in the center of the pouch can obliterate canal formation. Otic-specific knockout of bone morphogenetic protein 2 (*Bmp2*) results in absence of all three semicircular canals; however, the common crus and ampullae housing the sensory tissue (crista) are intact. The lack of *Bmp2* causes *Ntn1* (which encodes netrin 1), which is required for canal resorption, to be ectopically expressed at the canal rim. Ectopic *Ntn1* results in reduction of *Dlx5* and *Lmo4*, which are required for rim formation. These phenotypes can be partially rescued by removing one allele of *Ntn1* in the *Bmp2* mutants, indicating that Bmp2 normally negatively regulates *Ntn1* for canal formation. Additionally, non-resorption of the canal pouch in *Ntn1^−/−^* mutants is partially rescued by removing one allele of *Bmp2*. Thus, reciprocal inhibition between Bmp2 and netrin 1 is involved in canal formation of the vestibule.

## Abstract (precis)

**Summary:** *Bmp2*-conditional mutant analyses support the hypothesis that presumptive crista induces canal genesis zone in the canal pouch to express *Bmp2*, which promotes canal formation by restricting *Ntn1* expression to the resorption domain.

## INTRODUCTION

**Fig. 1.**
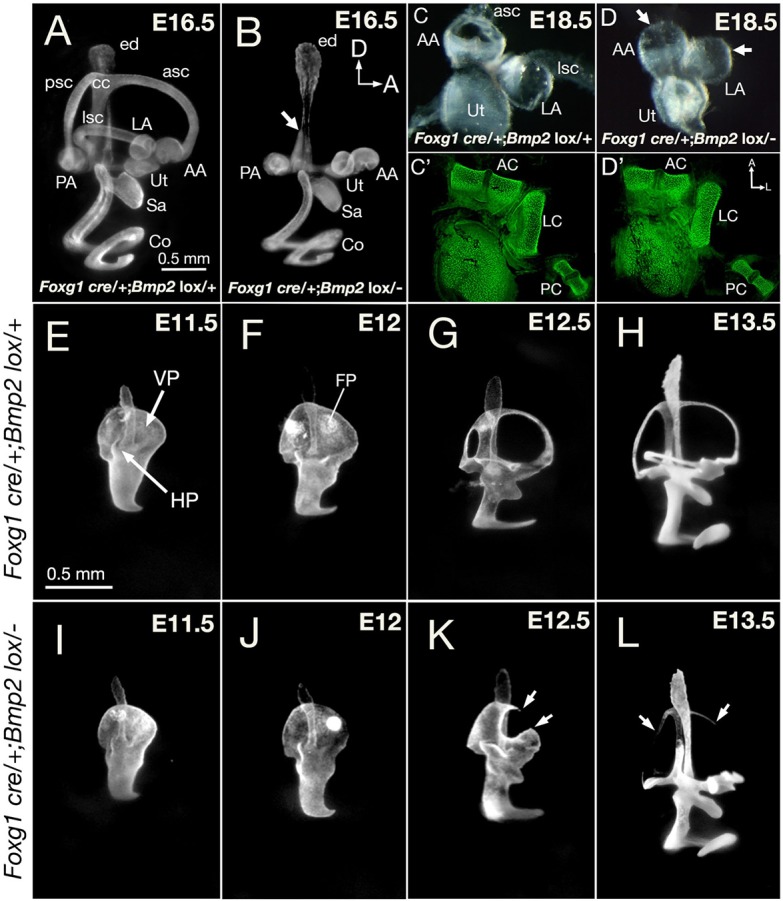
**Phenotypes of *Foxg1^cre/+^; Bmp2^lox/−^* inner ears.** (A,B) Paint-filled *Foxg1^cre/+^; Bmp2^lox/+^* (A) and *Foxg1^cre/+^; Bmp2^lox/−^* (B) inner ears at E16.5. (B) The conditional mutants are missing all three semicircular canals, and the common crus is thinner (arrow); however, the endolymphatic duct, utricle, saccule and cochlear duct appear normal. (C,D) Dissected membranous labyrinths of the utricle and the anterior and lateral ampullae in heterozygous controls (C) and in conditional mutants (D) at E18.5. Mutant ampullae have no canal opening (D, arrows) but the cristae within appear intact based on phalloidin staining of sensory hair cells (C′,D′). (E-L) Semicircular canal development in *Foxg1^cre/+^; Bmp2^lox/+^* (E-H) and *Foxg1^cre/+^; Bmp2^lox/−^* (I-L) ears between E11.5 and E13.5. (E-H) The vertical and lateral canal pouches in heterozygous controls are apparent at E11.5, with fusion plates emerging by E12 and followed by resorption. Canals reach their adult pattern by E13.5, but the size of the canals continues to increase after this age. (I-L) The *Foxg1^cre/+^; Bmp2^lox/−^* canal pouch (I) is slightly smaller than controls (E) at E11.5, but reduction in size is clear by E12 (J). (K) At E12.5, an opening is observed in the anterior region of the vertical canal pouch (arrows). (L) By E13.5, only remnants of the three canals are evident (arrows). AA, anterior ampulla; AC, anterior crista; asc, anterior semicircular canal; CC, common crus; Co, cochlea; ed, endolymphatic duct; FP, fusion plate; HP, horizontal canal pouch; LA, lateral ampulla; LC, lateral crista; lsc, lateral semicircular canal; PA, posterior ampulla; PC, posterior crista; psc, posterior semicircular canal; VP, vertical canal pouch; Sa, saccule; Ut, utricle. Orientations: A, anterior; D, dorsal. Orientation in B also applies to A,E-L. Orientation in D′ applies to C-D. Scale bars: 0.5 mm in A for A,B; 0.5 mm in E, for E-L.

The three semicircular canals of the inner ear and their associated sensory tissues, the cristae, are responsible for detecting angular head movements. Morphogenesis of the semicircular canals begins when the epithelia from the dorsal and lateral regions of the developing otocyst extend outward. These two epithelial extensions form pouches containing cavities that are continuous with the lumen of the otocyst (Fig. 1E-H, Fig. S1; Fekete et al., 1997; Martin and Swanson, 1993). Vertical (dorsal) and horizontal canal pouches are converted into separate canals when the two epithelial layers that create the walls of each pouch merge towards each other in the central region of each prospective canal and then fuse, forming fusion plates in response to mesenchymal proliferation and epithelial signaling via molecules such as netrin 1 and fibroblast growth factor 9 (Fgf9) (Pirvola et al., 2004; Salminen et al., 2000). Epithelial cells within the fusion plate resorb via apoptosis and/or retract towards the edge of the fusion plate (Fekete et al., 1997; Martin and Swanson, 1993). In contrast, the epithelia layers at the rim and at the center of the vertical canal pouch do not fuse, thus creating a continuous network of hollow tubes that connect to the remainder of lumen of the ear, forming the anterior and posterior canals that are joined by the common crus in the center. Similar resorption events occur in the horizontal canal pouch, except only the rim region of this pouch is preserved to form the lateral canal.

Based on fate mapping and gene expression analyses in the chicken inner ear, it was hypothesized that signaling molecules in the prospective sensory crista induce the adjacent tissue at the rim of the canal pouch to become the canal genesis zone that gives rise to the canals, as well as to some of the cells in the common crus (Fig. S1; Chang et al., 2004; Wu and Kelley, 2012). On the other hand, cells in the rest of the canal pouch largely give rise to the common crus or are resorbed. This unusual growth pattern of the canal pouch is thought to be mediated by Fgfs such as Fgf10, which is secreted from the prospective crista and induces *Bmp2* expression in the canal genesis zone (Chang et al., 2004). It is not clear, however, whether this mechanism proposed in the chicken is direct and/or conserved. Other evidence in support of the role for Fgf signaling in Bmp2-mediated canal formation comes from studies showing that *Fgf10* has a similar expression pattern in the presumptive cristae in mice (Pauley et al., 2003; Pirvola et al., 2000), and all three canals are missing in *Fgf10* knockout mice (Ohuchi et al., 2005; Pauley et al., 2003). While this canal phenotype is consistent with the model of Fgfs secreted in the cristae mediating canal pouch formation, it is still not clear whether this effect of Fgf10 in the mouse inner ear is direct because knockouts of other genes expressed in the presumptive cristae, such as *Bmp4* and *Jag1* (which encodes a ligand of the Notch signaling pathway), resulted in similar canal phenotypes (Chang et al., 2008; Kiernan et al., 2006; Morrison et al., 1999). Nevertheless, if the canal genesis zone and Bmp2 are involved in canal formation in mammals in a similar manner to that described in chicken (Chang et al., 2004), then Bmp2 should be required for the formation of the canals but not the ampullae or the common crus in mammals. We tested this hypothesis by generating conditional knockout mice in which *Bmp2* expression is absent in the developing mouse inner ear. The *Bmp2* conditional mutant phenotypes support a model in which crista mediates canal formation via the induction of a canal genesis zone and Bmp2 is an important effector of this zone. Furthermore, our results show that one of the mechanisms whereby Bmp2 promotes canal formation is by the restriction of *Ntn1* expression to the resorption domain.

## RESULTS

### Absence of semicircular canals in *Foxg1^cre/+^; Bmp2^lox/−^* embryos

*Bmp2* knockout mice die during early embryogenesis prior to sufficient inner ear development (Zhang and Bradley, 1996). Therefore, we generated conditional knockout of *Bmp2* in the inner ear using *Foxg1^cre^* mice that start to express *cre* in the invaginating otic placode (Hébert and McConnell, 2000). qRT-PCR results of vestibular tissue at E11.75 confirmed the absence of *Bmp2* transcripts in the conditional mutants compared with heterozygous controls (Fig. S2). Analyses of paint-filled *Foxg1^cre/+^; Bmp2^lox/−^* inner ears indicate the absence of all three semicircular canals, although a thin common crus is evident at embryonic day (E) 15.5 or older (Fig. 1B, arrow; *n*=12; compare with heterozygotes in Fig. 1A). Compared with controls (Fig. 1C,C′), the three ampullae in mutants are intact with no canal opening (Fig. 1D, arrows), and the cristae within the ampullae appear normal based on phalloidin staining of sensory hair cells (Fig. 1D′). This combination of absent canals with retained common crus and ampullae is consistent with the postulated role of *Bmp2* in chicken and zebrafish studies (Chang et al., 2004; Hammond et al., 2009). Other inner ear structures, such as the endolymphatic duct, utricle, saccule and cochlear duct, are indistinguishable from controls (Fig. 1A,B).

Semicircular canals develop between E11.5 and E13.5 in control mice (Fig. 1E-H). The two epithelial outpockets, the vertical and horizontal canal pouches, are evident by E11.5 (Fig. 1E, VP and HP). Over time, the opposing epithelia in the center region of the canal pouch merge towards each other to form a fusion plate (Fig. 1F, FP) that undergoes epithelial resorption, leaving the remaining epithelial cells at the rim to form the tube-shaped canal (Fig. 1G,H). Resorption takes place in the prospective anterior canal first, followed sequentially by the posterior and lateral canals (Fig. 1F,G; Morsli et al., 1998). All three canals are formed by E13.5, and they only increase in size after this age (Morsli et al., 1998).

Compared with controls (Fig. 1E,F), *Foxg1^cre/+^; Bmp2^lox/−^* canal pouches are present, although slightly reduced in size, at E11.5, with the size reduction becoming more apparent by E12 (Fig. 1I,J, E11.5-12, *n*=16). By E12.5, part of the anterior rim is missing in the vertical canal pouch (Fig. 1K, arrows, *n*=8), and there is no anterior canal. Only remnants of the three canals are evident by E13.5 (Fig. 1L, arrows, *n*=18). These results indicate that the canal defects observed in the *Bmp2* conditional mutants are initiated early during canal development and are not a result of canal degeneration subsequent to formation.

### Expression pattern of *Bmp2* in developing semicircular canals

**Fig. 2.**
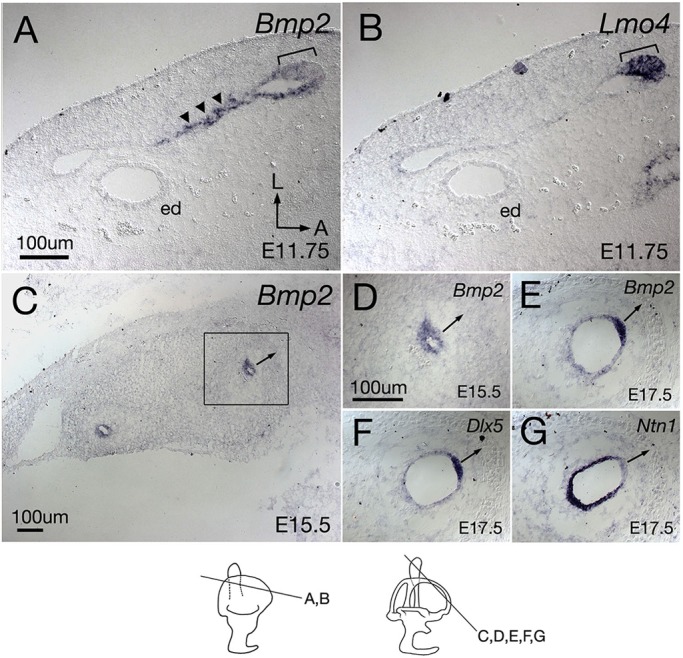
***Bmp2* expression during semicircular canal formation.** (A) A mid-section through the vertical canal pouch at E11.75. *Bmp2* is broadly expressed in the fusion plate (arrowheads) and the rim region of the canal pouch (bracket). (B) Adjacent section to A showing *Lmo4* is exclusively expressed in the rim domain (bracket). (C) At E15.5, *Bmp2* is expressed in the entire canal epithelium. (D) A high-magnification view of the rectangle in C. (E-G) Adjacent sections of the anterior semicircular canal at E17.5 probed for *Bmp2* (E), *Dlx5* (F) and *Ntn1* (G). *Bmp2* is restricted to the outer rim of the canal (E), overlapping with the *Dlx5* domain (F). In contrast, *Ntn1* shows a complementary expression pattern with *Bmp2* (E) and *Dlx5* (G). Arrows are pointing away from the outer rim of the canal. ed, endolymphatic duct. Orientations: A, anterior; L, lateral. Orientation in A also applies to B-G. Scale bars: 100 μm in A for A,B; 100 μm in C; 100 μm in D for D-G.

To address the role of *Bmp2* in canal formation, we investigated its normal expression pattern in the developing inner ear and compared its expression pattern with that of two other genes that are required for canal formation, *Lmo4* (which encodes a LIM-only domain transcription regulator) and *Dlx5* (which encodes distaless-related 5 homeodomain transcription factor) (Deng et al., 2010; Merlo et al., 2002). Both *Lmo4* and *Dlx5* are activated prior to canal pouch formation in the lateral region of the otocyst (Deng et al., 2010; Merlo et al., 2002). By the time the canal pouches are evident at E11.5, the expression domains of *Lmo4* (Fig. 2B) and *Dlx5* (not shown) are restricted to the rim of the canal pouch. In contrast, *Bmp2* transcripts are only detectable after canal pouches are morphologically evident and not prior. At E11.75, *Bmp2* is expressed strongly in the fusion plate (Fig. 2A, arrowheads) and more weakly at the rim of the canal pouch (Fig. 2A, bracket) that is positive for *Lmo4* (Fig. 2B). After canal formation, *Bmp2* is initially expressed in the entire canal epithelium at E15.5 (Fig. 2C,D), but its expression domain becomes restricted to the outer rim of the canals over time (Fig. 2E), where it overlaps with the expression domain of *Dlx5* (Fig. 2F) but is complementary to that of *Ntn1*, which is expressed in the inner rim of canals (Fig. 2G).

### Decreased cell proliferation at the rim of *Foxg1^cre/+^; Bmp2^lox/−^* inner ears

**Fig. 3.**
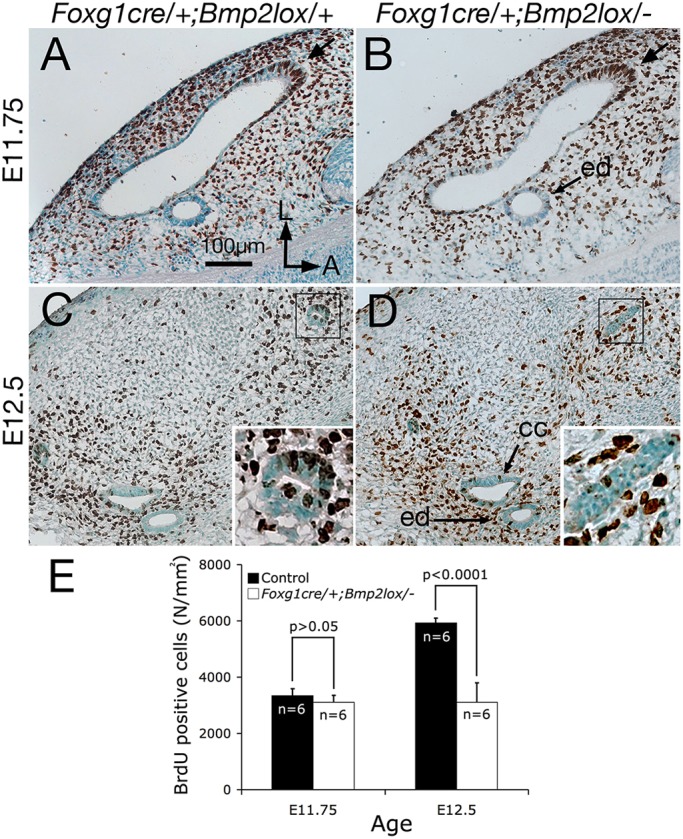
**Decreased cell proliferation in the *Foxg1^cre/+^; Bmp2^lox/−^* canal pouch.** (A-D) BrdU labeling of *Foxg1^cre/+^; Bmp2^lox/+^* (A,C) and *Foxg1^cre/+^;* *Bmp2^lox/−^* (B,D) canal pouches visualized using alkaline phosphatase-conjugated secondary antibodies at E11.75 (A,B) and E12.5 (C,D) to label proliferating cells. At E11.75, the number of BrdU-positive cells per epithelial area is not reduced in *Foxg1^cre/+^;* *Bmp2^lox/−^* inner ears (B), compared with heterozygous controls (A,E; *P*>0.05). By E12.5, *Foxg1^cre/+^;* *Bmp2^lox/−^* inner ears (D) show reduced cell proliferation, compared with controls (C,E; *P*<0.0001). (E) Quantification of BrdU-labeled cells between *Bmp2* heterozygous and conditional knockout canal pouches at E11.75, and anterior and posterior canals at E12.5. Orientation in A applies to B-D. CC, common crus; ed, endolymphatic duct. Scale bar: 100 μm.

The early reduction in the size of the mutant canal pouch suggests that Bmp2 may be required for epithelial proliferation. Therefore, we examined proliferation in *Foxg1^cre/+^; Bmp2^lox/−^* inner ears using BrdU. The majority of cell proliferation of the canal pouch occurs at the rim (Fig. 3A,C, arrow), which is similar to observations reported in the chicken (Chang et al., 1999, 2002). To quantify BrdU-positive cells, three representative sections in the vertical canal pouch at E11.75 (*n*=3 embryos) or the anterior and posterior canals at E12.5 (*n*=3 embryos), were selected from each ear and the average number of BrdU-positive cells per canal epithelial area was calculated as described in the Materials and Methods. No statistical significant difference was observed in the percentages of BrdU-positive epithelial cells between *Foxg1^cre/+^; Bmp2^lox/−^* and heterozygous control pouches at E11.75 (Fig. 3A,B,E; *P*=0.0736). Despite the absence of a distinct anterior canal, based on *Foxg1^cre/+^; Bmp2^lox/−^* paint-filled ears at E12.5 (Fig. 1K), cryo-sections reveal some residual canal epithelia without a lumen (Fig. 3D, inset). These canal tissues show a 52% reduction in cell proliferation per unit area (Fig. 3D,E; *P*=0.000003) compared with heterozygous controls (Fig. 3C,E). In contrast, we did not observe a significant difference in BrdU-positive cells between endolymphatic ducts of controls and mutants (*P*=0.2182).

As aberrant cell death could also affect normal canal pouch outgrowth, we investigated the role of apoptosis in contributing to the mutant canal phenotype using TUNEL. No obvious difference in the number or distribution of apoptotic cells between control and conditional mutant vestibules at E11.75 and E12.5 was observed (Fig. S3, *n*=6). Together, these results suggest that Bmp2 is required for maintaining cell proliferation in the canal pouch during canal formation.

### Downregulation of pSmad1/5/8, *Dlx5* and *Lmo4* at the rim of *Foxg1^cre/+^; Bmp2^lox/−^* canals

**Fig. 4.**
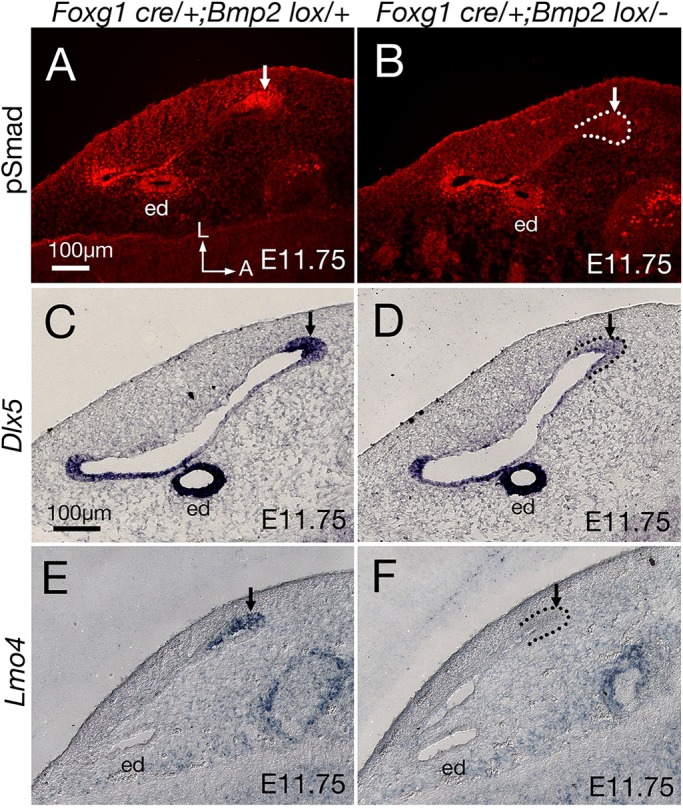
**Downregulation of genes expressed in the rim of the *Foxg1^cre/+^; Bmp2^lox/−^* canal pouch.** (A,C,E) pSmad (A), *Dlx5* (C) and *Lmo4* (E) are expressed at the rim of the anterior canal pouch in heterozygous controls (arrow) at E11.75. (B,D,F) *Foxg1^cre/+^;* *Bmp2^lox/−^* inner ears show reduced expression of pSmad 1/5/8 (B), *Dlx5* (D) and *Lmo4* (F) in the rim domain (arrow). For easier visualization, parts of the outer rim of the canal epithelia are outlined with dotted lines. Orientation in A also applies to B-F. ed, endolymphatic duct. Scale bars: 100 μm in A for A,B; 100 μm in C for C-F.

Reduction of epithelial growth and/or increased resorption can lead to the failure of canal formation. As *Bmp2* is broadly expressed in the canal pouch, including both the rim (the prospective canal region) and the resorption domain, it is possible that Bmp2 may have dual roles in canal formation: promoting growth and inhibiting resorption. To investigate the mechanism of Bmp2 function, we focused our analyses on the anterior canal. We asked which cells in the anterior part of the vertical canal pouch are responsive to Bmp2 by investigating the presence of phosphorylated Smad 1/5/8 (pSmad), a downstream transducer of Bmp signaling (ten Dijke et al., 2000). At E11.75, pSmad immunoreactivity is detected strongly at the rim of the canal pouch, as well as in the endolymphatic duct epithelium and in the mesenchymal cells surrounding the endolymphatic duct (Fig. 4A). In *Foxg1^cre/+^; Bmp2^lox/−^* inner ears, there is a marked reduction of pSmad immunoreactivity in the anterior rim of the canal pouch (Fig. 4B; arrow), suggesting that the primary site of Bmp2 action is at the rim of the canal pouch domain. Consistently, both *Dlx5* and *Lmo4*, which are expressed in the canal rim, are also downregulated at the rim of the mutant canal pouch (Fig. 4D,F, arrow), compared with heterozygous controls (Fig. 4C,E). The downregulated *Dlx5* and *Lmo4* expression patterns and the decreased proliferation observed (Fig. 3) are consistent with the demonstrated function of *Dlx5* and *Lmo4* in canal formation (Deng et al., 2010; Merlo et al., 2002).

### Expansion of the *Ntn1* expression domain in *Foxg1^cre/+^; Bmp2^lox/−^* canal pouches

**Fig. 5.**
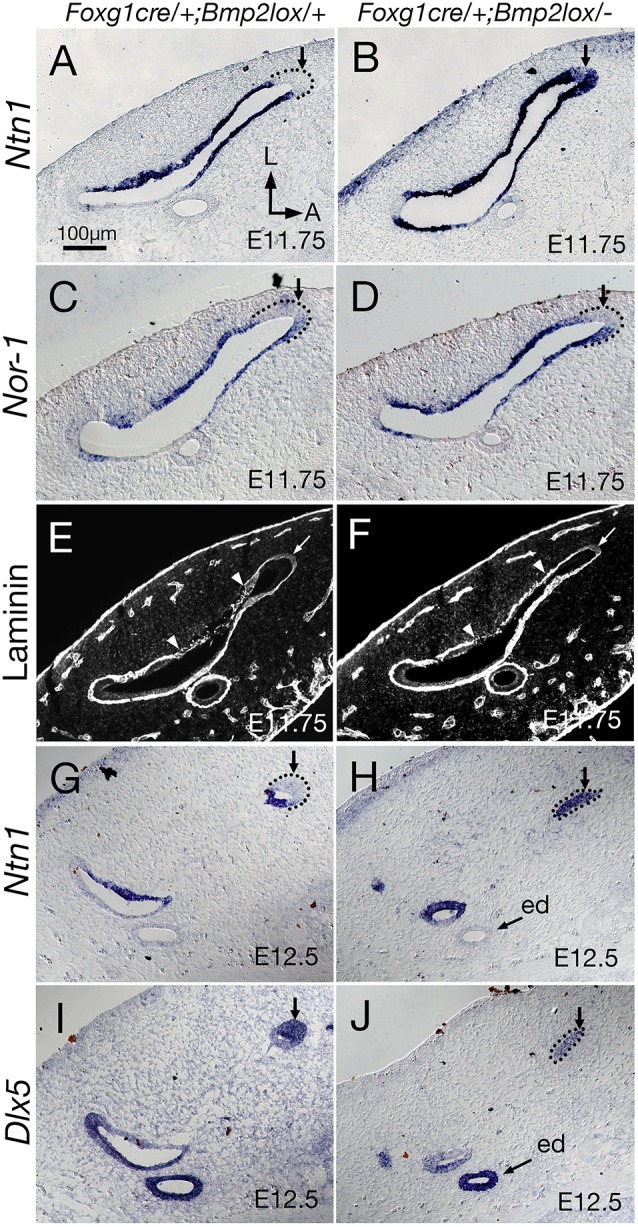
**Expansion of *Ntn1* expression to the rim of *Foxg1^cre/+^; Bmp2^lox/−^* canal pouch.** (A-D) Adjacent sections probed for *Ntn1* (A,B) and *Nor-1* (C,D) transcripts at E11.75. (A) *Ntn1* is expressed in the canal pouch but not the rim of *Foxg1^cre/+^;* *Bmp2^lox/+^* controls, whereas *Ntn1* expression is expanded around the rim of *Foxg1^cre/+^;* *Bmp2^lox/−^* canal pouches (B, arrow). (C) *Nor-1* expression in heterozygous controls is similar to *Ntn1*, and is not expressed at the rim (arrow). (D) Unlike *Ntn1*, the rim of the mutant canal pouch is devoid of *Nor-1* expression (arrow). (E,F) Anti-laminin antibody staining of the basement membrane at E11.75, which starts to show intermittent staining pattern in the lateral side of the fusion plate in both controls (E) and mutants (F) (arrowheads), shown in grayscale. No obvious difference in staining pattern between controls and mutants (arrows) is observed at the rim of the canal pouch. (G-J) Expression patterns of *Ntn1* (G,H) and *Dlx5* (I,J) of *Bmp2* heterozygous controls (G,I) and conditional mutants (H,J) at E12.5. (G,I) The complementary expression domain of *Ntn1* and *Dlx5* in the canal is apparent at E12.5. (H,J) In remnants of *Foxg1^cre/+^;* *Bmp2^lox/−^* canals, *Ntn1* expression is strong (H, arrow) but *Dlx5* is weaker (J, arrow). ed, endolymphatic duct. Orientation in A also applies to B-J. Scale bar: 100 μm.

As *Bmp2* is also expressed in the resorption domain of the canal pouch (Fig. 2A), we examined expression of some of the genes that are specifically expressed in the resorption domain in the *Bmp2* conditional mutants. Both *Ntn1* and *Nor-1* (*Nr4a3*) are expressed in the resorption domain of the canal pouch, while the rim is devoid of hybridization signals (Fig. 5A,C, arrow; Ponnio et al., 2002; Salminen et al., 2000). After canal formation, these genes are expressed in the inner rim of canals (Fig. 5G; Ponnio et al., 2002; Rakowiecki and Epstein, 2013). Netrin 1 is required for the resorption process in mice as the fusion plate fails to develop and no resorption occurs in *Ntn1* knockout mice (Salminen et al., 2000). The function of *Nor-1* in canal formation is less clear. Inner ears of *Nor-1* knockouts display canals with thinner calibers, which could be caused by reduced proliferation as well as mis-regulated resorption (Ponnio et al., 2002). In *Foxg1^cre/+^; Bmp2^lox/−^* inner ears, the size of the canal rim appears smaller and the *Ntn1* expression domain is expanded around the edge of the anterior rim (Fig. 5B, arrow). In contrast to *Ntn1* expression (Fig. 5B), the anterior rim of the mutant canal pouch remains devoid of *Nor-1* expression (Fig. 5D, arrow) similar to heterozygous controls (Fig. 5C).

By E12.5, remnant anterior canal tissues of *Foxg1^cre/+^; Bmp2^lox/−^* inner ears lose the normal complementary expression patterns of *Ntn1* and *Dlx*5 observed in controls (Fig. 5G,I; Rakowiecki and Epstein, 2013), showing, instead, prominent *Ntn1* expression and diffuse *Dlx5* expression throughout the epithelium (Fig. 5H,J). Although this strong expression of *Ntn1* at E12.5 in *Foxg1^cre/+^; Bmp2^lox/−^* inner ears is consistent with the earlier upregulation of *Ntn1* observed at the rim of the canal pouch at E11.75 (Fig. 5B), it is not clear whether these changes in the *Ntn1* expression pattern in the mutant canal pouch represent an actual upregulation of *Ntn1* or simply the appearance of upregulation due to a loss of canal rim tissue. Notably, qRT-PCR analyses of the dorsal region of the inner ear, which included the canal pouch, the endolymphatic duct and some surrounding mesenchyme, did not show a significant change in the levels of *Ntn1*, *Dlx5*, *Nor-1* and *Lmo4* transcripts, although the predicted downregulation of *Bmp2* between conditional knockouts and heterozygous controls was detected (Fig. S2).

### Upregulated *Ntn1* expression without expedited basement membrane breakdown in *Foxg1^cre/+^; Bmp2^lox/−^* canal pouches

**Fig. 6.**
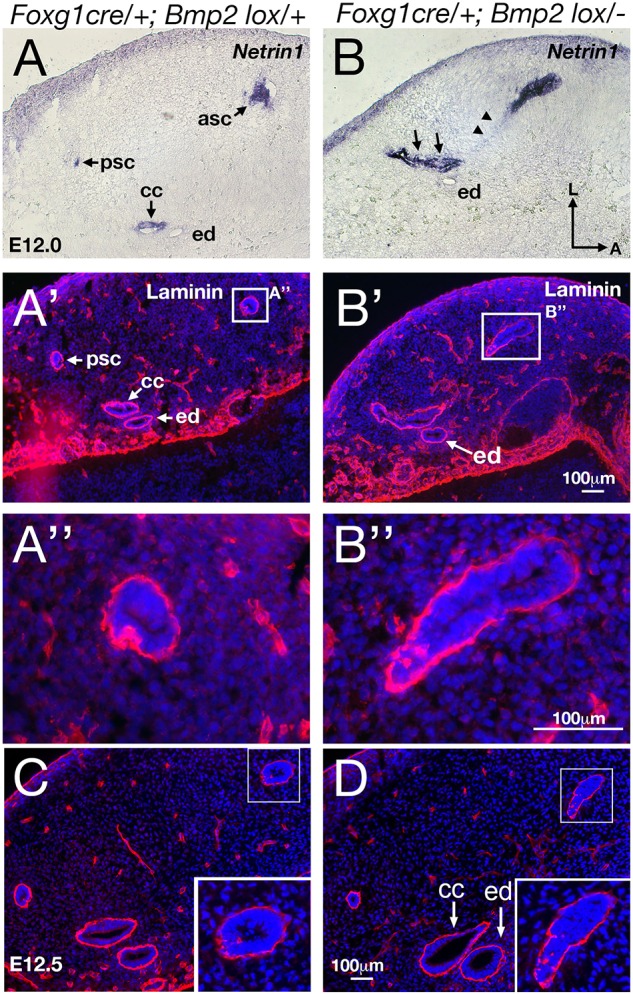
**No premature basement membrane breakdown in *Bmp2* conditional knockout mutants.** Adjacent sections of *Foxg1^cre/+^;* *Bmp2^lox/+^* (A-A″,C) and *Foxg1^cre/+^;* *Bmp2^lox/−^* (B-B″,D) canals/canal pouches at E12 (A-B″) and E12.5 (C,D) that were processed for *Ntn1* (A,B) and anti-laminin antibody staining (A′,A″,B′,B″,C,D). At E12, the anterior and posterior canals (asc and psc) in the control show intact basement membrane (A′,A″) with *Ntn1* expressed in the inner rim of each canal (A). In contrast, the *Bmp2* mutant (B-B″) shows upregulated *Ntn1* expression at the canal rim (B) but its basement membrane appears intact (B″). In comparison with the control (A-A″), resorption is not complete in the mutant (B-B″, arrows and arrowheads). There is no distinguishable posterior canal (psc) or common crus (arrows in B), and remnant *Ntn1* expression is observed in the resorption domain of the anterior canal pouch region (arrowheads in B). By E12.5, canal resorption seems complete and comparable between the control (C) and mutant (D), but the remnant anterior canal in the mutant (inset in D) looks similar to the one at E12.0 (B″) with an intact basement membrane. asc, anterior semicircular canal; cc, common crus; ed, endolymphatic duct; psc, posterior semicircular canal. Scale bar: 100 μm.

Within the normal resorption domain, the basement membrane breaks down and epithelial cells disappear or retract towards the edge of the domain (Martin and Swanson, 1993). The failure of basement membrane breakdown is one of the phenotypes associated with loss of *Ntn1* (Salminen et al., 2000). Therefore, we investigated whether the change in *Ntn1* expression at the rim of the mutant canal pouch (Fig. 5B) is associated with abnormal breakdown of the basement membrane. Anti-laminin staining shows no obvious difference between controls (Fig. 5E) and mutants (Fig. 5F) in the staining pattern at the rim (arrow) or resorption domain (arrowheads) of the E11.75 canal pouches. We did not observe any significant differences in the extent of basement membrane breakdown between the mutants and their littermate controls (ratio of disrupted basement membrane length between controls and mutants=1.09, s.d.=0.16, *n*=3). However, fusion plate formation followed by basement membrane breakdown is an ongoing dynamic process, so changes in basement membrane breakdown may not be apparent at an early age (E11.75). Hence, we analyzed late stage canal resorption, reasoning that increased basement membrane breakdown should be accumulative and resorption should be more advanced in the mutants than in controls at later stages. By contrast, we found that resorption is more advanced in controls at E12. When some of the canals are already apparent in littermate controls (Fig. 6A-A″), *Bmp2* mutants show upregulated *Ntn1* expression and presence of a lumen at the canal rim but resorption is not complete (Fig. 6B-B″, *n*=3), suggesting that there is no premature or enhanced membrane breakdown caused by the upregulated *Ntn1* expression in the mutants. By E12.5, this *Ntn1*-basement membrane relationship is maintained in the canal even though the lumen is collapsed (Figs 5H and 6D), indicating that upregulated *Ntn1* expression is not correlated with basement membrane breakdown in the *Bmp2* mutants. Together, our results suggest that if the change in *Ntn1* expression causes the demise of canal formation in the *Bmp2* mutants, this effect is not mediated by premature or expedited basement membrane breakdown. The persistence of *Ntn1* expression in the inner rim of normal canals (Figs 5G and 6A) also lends support to the idea that the presence of *Ntn1* is not necessarily correlated with epithelial resorption.

### Reciprocal negative regulation between *Bmp2* and *Ntn1* in canal formation

**Fig. 7.**
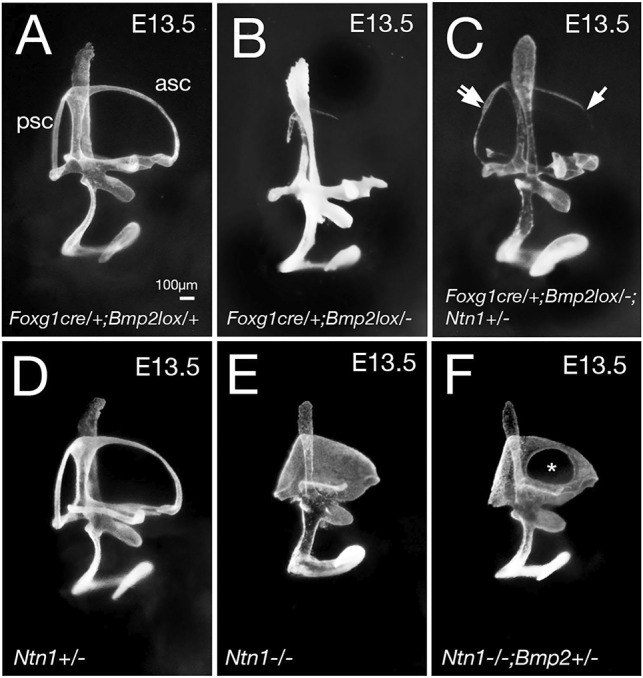
**Reciprocal inhibition of *Bmp2* and *Ntn1* function in canal formation.** (A-C) Paint-filled *Foxg1^cre/+^; Bmp2^lox/+^* (A, control), *Foxg1^cre/+^;* *Bmp2^lox/−^* (B) and *Foxg1^cre/+^;* *Bmp2^lox/−^;* *Ntn1^+/−^* (C) inner ears at E13.5. (B) *Foxg1^cre/+^;* *Bmp2^lox/−^* inner ears lack the three semicircular canals. This canal phenotype of *Foxg1^cre/+;^ Bmp2^lox/−^* ears is partially rescued by eliminating one allele of *Ntn1* (C). In *Foxg1^cre/+^;* *Bmp2^lox/−^;* *Ntn1^+/−^* inner ears (C; *P*<0.0001), the anterior canal is longer (single arrow) and the posterior canal is intact (double arrows). (D-F) Paint-filled *Ntn1^+/−^* (D), *Ntn1^−/−^* (E) and *Ntn1^−/−^;* *Bmp2^+/−^* (F) inner ear at E13.5. (D) *Ntn1^+/−^* inner ears are normal. (E) *Ntn1^−/−^* inner ears show non-resorption of the canal pouches; this phenotype is partially rescued for the anterior canal in the absence of one allele of *Bmp2* (F, asterisk; *P*<0.05). Scale bar: 100 μm.

**Fig. 8.**
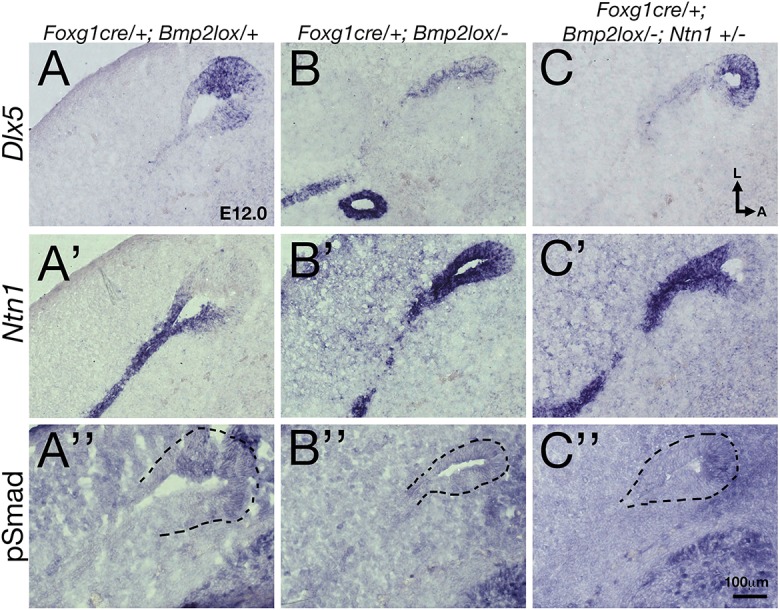
**Canal identity is recovered in *Foxg1^cre/+^;Bmp2^lox/−^;Ntn1^+/−^* inner ears.** Adjacent sections of the canal pouch in *Foxg1^cre/+^; Bmp2^lox/+^* (A-A″), *Foxg1^cre/+^;* *Bmp2^lox/−^* (B-B″) and *Foxg1^cre/+^;* *Bmp2^lox/−^;* *Ntn1^+/−^* (C-C″) inner ears at E12 that were processed for *Dlx5* (A,B,C), *Ntn1 in situ* hybridization (A′,B′,C′) and anti-pSmad antibody staining (A″,B″,C″). In *Foxg1^cre/+^;* *Bmp2^lox/−^;* *Ntn1^+/−^* ears (C-C″), the upregulation of *Ntn1* (B′) and the reduction of *Dlx5* (B) and pSmad (B″) observed in *Foxg1^cre/+^;* *Bmp2^lox/−^* canal rims are restored to the expression patterns seen in controls (A-A″). Dotted lines outline the rim of the canal pouch. Scale bar: 100 μm.

To further determine whether the upregulated *Ntn1* expression in *Bmp2* conditional mutants has any significant contribution to the loss of canal phenotype, we conducted a genetic experiment by generating *Foxg1^cre/+^; Bmp2^lox/−^* embryos that lack one allele of *Ntn1*. We reasoned that if Bmp2 mediates its function by negatively regulating *Ntn1* expression in the canal pouch, the canal phenotype in the *Bmp2* conditional mutants might be partially rescued by removing one allele of *Ntn1*. Indeed, in *Foxg1^cre/+^; Bmp2^lox/−^;* *Ntn1^+/−^* inner ears, longer partial anterior canals (>50% of control length, *P*<0.0001) and increased frequency of intact posterior canals (*P*<0.0001) are evident, compared with *Foxg1^cre/+^; Bmp2^lox/−^* inner ears (Fig. 7A-C, Table 1). *Ntn1^+/−^* canals are normal (Fig. 7D; Abraira et al., 2008). Gene expression patterns of the anterior canal rim in *Foxg1^cre/+^; Bmp2^lox/−^;* *Ntn1^+/−^* inner ears (Fig. 8C-C″) are similar to controls (Fig. 8A-A″) but different from those in *Foxg1^cre/+^; Bmp2^lox/−^* ears (Fig. 8B-B″). The rescued canals show no ectopic *Ntn1* expression but pSmad and *Dlx5* are present at the rim of the canal pouch (Fig. 8C-C″, *n*=7). These results provide genetic and cellular evidence that Bmp2 normally negatively regulates *Ntn1* expression at the canal rim, which directly or indirectly affects the rim identity.

**Table 1.**
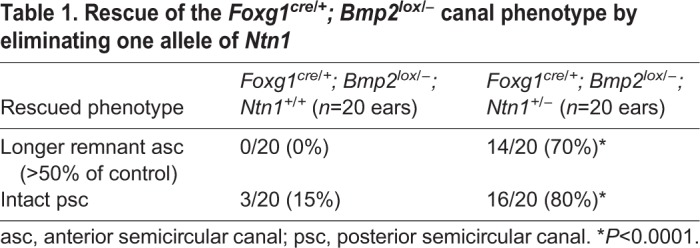
**Rescue of the *Foxg1^cre/+^; Bmp2^lox/−^* canal phenotype by eliminating one allele of *Ntn1***

**Fig. 9.**
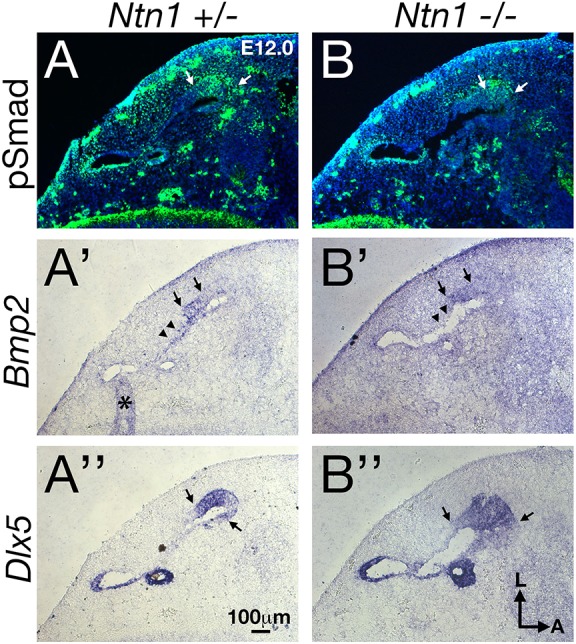
**Gene expression analyses of *Ntn1^−/−^* canal pouch.** Adjacent sections of the canal pouch of *Ntn1^+/−^* (A-A″) and *Ntn1^−/−^* (B-B″) embryos at E12 that were processed for anti-pSmad antibody staining (A,B), and *Bmp2* (A′,B′) and *Dlx5* (A″,B″) *in situ* hybridization. Arrows delineate the approximate borders of strong expression domains and an asterisk represents an artifact created by a fold in the tissue. There is no obvious change in the expression domain of pSmad, *Bmp2* and *Dlx5* in the *Ntn1^−/−^* compared with controls. Arrowheads indicate the fusion plate region, in which *Bmp2* expression in the mutant may be reduced (B′). Scale bar: 100 μm.

Next, to investigate whether there is a reciprocal inhibition of Ntn1 by Bmp2, we generated *Ntn1*^−/−^ mice in a *Bmp2^+/−^* background. The extent of canal non-resorption is variable in the *Ntn1* mutants (Table 2); however, in the presence of only one allele of *Bmp2*, the frequency of a distinct anterior canal increases from 57% to 89% (Fig. 7F, Table 2; *P*=0.0198). These results indicate that netrin 1 also negatively regulates Bmp2 functions. To address whether Ntn1 normally inhibits Bmp2 function by suppressing *Bmp2* expression, we investigated whether *Bmp2* expression and pSmad are upregulated in *Ntn1^−/−^* ears during canal formation. Although the *Ntn1* mutants always show a delay in canal development relative to the littermate controls, we did not observe an obvious upregulation of *Bmp2* expression domain or pSmad immunostaining in *Ntn1* knockouts, as might be predicted by the genetic results (Fig. 9A,A′,B,B′, arrows, *n*=12 ranging from E11.75-12.5). In fact, there may be a reduction in *Bmp2* expression in the center of the canal pouch (Fig. 9B′, arrowheads, *n*=3/11) but this reduction was not consistently observed among all the specimens. In addition, there is no obvious expansion of the canal rim identity based on the *Dlx5* expression pattern (Fig. 9A″,B″, arrows, *n*=10). These results suggest that Ntn1 is unlikely to mediate canal resorption by inhibiting *Bmp2* expression or restricting *Dlx5* expression to the rim, and the role of Ntn1 in negatively regulating Bmp2 functions remains to be determined.

**Table 2.**
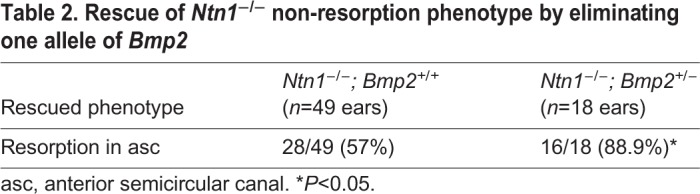
**Rescue of *Ntn1*^−/−^ non-resorption phenotype by eliminating one allele of *Bmp2***

## DISCUSSION

### Conserved role of *Bmp2* in canal formation

Although not necessarily recognized at the time, the earliest results that implicated *Bmp2* in canal formation were conducted with chicken embryos, in which treatments with noggin (Nog, an antagonist of Bmp2 and Bmp4) or virus encoding *Nog* resulted in truncation or loss of inner ear canals (Chang et al., 1999; Gerlach et al., 2000). Subsequent studies (also conducted *in ovo*) proposed a model in which Fgfs secreted from the presumptive crista mediate canal formation by upregulating *Bmp2* in the canal pouch (Chang et al., 2004). More recently, *bmp2b* was shown to be required for maintaining canal structures in zebrafish (Hammond et al., 2009). Mutant *bmp2b* zebrafish have no canals but intact ampullae and common crus, similar to the mouse mutants reported here. Taken together, results in both zebrafish and mice strongly suggest that *Bmp2* has a conserved role in canal formation. Furthermore, our studies provide insights into the mechanisms of this Bmp2 function.

### Timing of the requirements for Bmp2 in canal formation

**Fig. 10.**
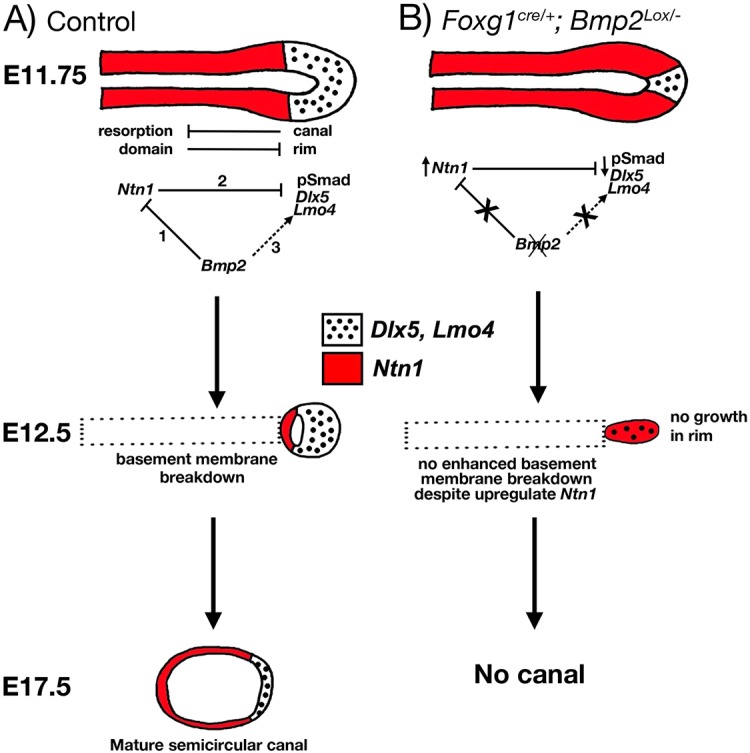
**Role of Bmp2 in semicircular canal formation.** (A) Normal canal development from E11.75 to E17.5. At E11.75, pSmad, *Dlx5* and *Lmo4* (black dots) are expressed in the rim of the canal pouch, whereas *Ntn1* (red) is expressed in the resorption domain. *Bmp2* is expressed in both of these domains of the canal pouch and it functions to restrict *Ntn1* expression to the resorption domain (1) and *Ntn1* indirectly restricts rim identity (2). In addition, the rim domain may be another target region of Bmp2 based on pSmad 1/5/8 staining (3). (B) In the absence of *Bmp2*, there is an expansion of the *Ntn1* expression to the rim, which results in a reduction in epithelial cell proliferation and downregulation of *Dlx5* and *Lmo4*, collectively producing the failure of canal formation. The dotted arrows represent postulated effects based on our genetic and gene expression analyses.

Both gene expression and phenotypic analyses of *Bmp2* conditional mutants indicate that Bmp2 has a slightly different role from other genes implicated in canal formation, such as *Dlx5*, *Hmx2*, *Hmx3* and *Lmo4* (Deng et al., 2010; Hadrys et al., 1998; Merlo et al., 2002; Wang et al., 2004, 1998). Most of these genes are broadly expressed in the otocyst initially and become restricted to the canal pouch by the time the pouch is morphologically evident. In the cases of *Dlx5* and *Lmo4*, their transcripts are restricted to the rim of the canal pouch (Fig. 10A). In contrast, *Bmp2* transcripts are detected robustly only after the canal pouches are established. Consistent with these expression patterns, knockout of *Dlx5*, *Hmx2*, *Hmx3* and *Lmo4* generated vestibular phenotypes that are more severe and variable than that of the *Bmp2* conditional mutants (Deng et al., 2010; Hadrys et al., 1998; Merlo et al., 2002; Wang et al., 2004, 1998). Severe phenotypes, such as a much-reduced size of the canal pouch or a complete lack of resorption reported in *Dlx5* and *Hmx* mutants, are not observed in the *Bmp2* conditional mutants and this is indicative of *Dlx5* and *Hmx* having an earlier and broader role than *Bmp2* in canal formation. Nevertheless, we show that the maintenance of some of these early genes, such as *Dlx5* and *Lmo4*, are dependent on Bmp2 function.

### Is Bmp2 the downstream mediator of crista signaling in canal formation?

Several lines of evidence in chicken studies suggest that Bmp2 is a downstream mediator of crista signaling. First, its expression domain coincides with the canal genesis domain identified in fate-mapping studies using a lipophilic dye (Chang et al., 2004). Second, manipulating Fgf functions in the prospective crista by implanting beads soaked with Fgf2 or an inhibitor of Fgf receptor, SU5402, to the canal pouch resulted in gain and loss of canal tissues that were preceded by up- and downregulation of *Bmp2* expression, respectively (Chang et al., 2004). Consistently, the loss of common crus with Fgf2 treatments can be rescued by the simultaneous presence of noggin (Chang et al., 2004). However, despite the evidence supporting a role for Bmp2 in mediating canal formation, other ligands expressed in the prospective crista, such as jagged 1 (Jag1) and Bmp4 also qualify as candidates for canal induction based on studies in both chicken and mice (Chang et al., 2008; Kiernan et al., 2006). Furthermore, Fgfs, Jag1, Bmp4 and Wnts are dependent on each other for expression in the crista (Chang et al., 2008; Daudet et al., 2007; Rakowiecki and Epstein, 2013). Thus, further studies are required to determine whether Fgfs directly activate Bmp2 in the canal pouch.

Fate mapping studies in chicken suggest that the *Bmp2*-positive canal genesis zone contributes to most of the cells in the canals but contributes much less to the common crus (Chang et al., 2008). Although there is no direct evidence that a similar canal genesis zone exists in the mouse, the *Bmp2* expression domain in the mouse canal pouch, albeit broader, is similar to that of the chicken (Chang et al., 2004, 2008). Thus, it is interesting that the phenotypes obtained from the *Bmp2* conditional mutants (a fully penetrant phenotype of loss of canals with intact ampullae and a milder reduction in the size of the common crus) are entirely consistent with the canal genesis zone hypothesis formulated in chicken, suggesting that the establishment of a canal genesis zone is a conserved mechanism of canal formation.

### Mechanisms of Bmp2 in canal formation

Reciprocal inhibition between the rim and resorption domain of the canal pouch during canal formation have been well documented (Abraira et al., 2008; Huang et al., 2018). Our results indicate that broad expression of *Bmp2* in the canal pouch (Fig. 2) appears to be required for both of these domains during canal formation. First, our results suggest Bmp2 restricts the resorption process via negative regulation of *Ntn1* expression (Fig. 10A, #1 and #2). By reducing the gene dose of *Ntn1*, canals are partially recovered in the *Bmp2* conditional mutants (Fig. 7, Table 1). In the recovered canals, *Ntn1* is no longer upregulated at the canal rim and *Dlx5* and pSmad are expressed normally. These results suggest that Bmp2 negatively regulates *Ntn1* expression in the canal rim, which could indirectly affect rim identity (Fig. 10A, #1 and #2). However, if negative regulation of *Ntn1* is important for canal rim specification, one might expect the canal rim domain to be expanded in the *Ntn1^−/−^* canal pouch. This does not appear to be the case (Fig. 9). No obvious change in rim identity is observed based on pSmad and *Dlx5* expression. These results suggest that Bmp2 function is not mediated by regulation of *Ntn1* alone.

A possible additional function of Bmp2 is promoting canal rim formation where its transducer, pSmad, is expressed from canal pouch stage to canals (Figs 2 and 10A, #3). It is arguable that the presence of pSmad in the rescued canals of *Foxg1^cre/+^; Bmp2^lox/−^;* *Ntn1^+/−^* inner ears indicates that pSmad is not a direct readout of *Bmp2* and the site of Bmp2 action remains unclear (Fig. 8), and there is no direct experimental evidence that supports the notion that Bmp2 promotes canal rim formation. However, existing results from both chicken and zebrafish studies support the notion that *Bmp2* is also required for the continual growth of the canal after its initial formation and resorption is complete (Chang et al., 1999; Hammond et al., 2009). In summary, both of the above proposed mechanisms for Bmp2 function – in negatively regulating resorption via *Ntn1* (Fig. 10, #1 and #2) and in promoting rim identity via pSmad (Fig. 10, #3) – remain plausible.

### Negative regulation of *Ntn1* by *Bmp2* in canal formation

Ntn1 has many functions during embryogenesis. In addition to its well-established role in axonal guidance, it also mediates other cellular processes, such as cell migration, branching morphogenesis of lung and mammary gland, and canal resorption of the inner ear (Cirulli and Yebra, 2007; Lai Wing Sun et al., 2011). Its mechanism in canal resorption is not clear and does not seem to involve its known receptors in other systems (Abraira et al., 2008). *Ntn1* is thought to mediate canal resorption by mediating basement membrane breakdown of the resorption domain. The resorption role of Ntn1 in lateral canal formation is negatively regulated by Lrig3, an immunoglobulin superfamily protein (Abraira et al., 2008). In the absence of *Lrig3*, the *Ntn1* expression domain is expanded into the rim of the lateral canal pouch, which is accompanied by an expanded breakdown of the basement membrane. More recent gain-of-function studies of *Ntn1* in both chicken and mice suggested additional complexity regarding the role of Ntn1 in basement membrane breakdown (Nishitani et al., 2017). Although gain of *Ntn1* function in mice causes moderate effects of canal truncation, which presumably is due to ectopic canal resorption, gain of *Ntn1* function in chicken shows an increase in fusion plate formation without resorption. These results suggest that the effect of Ntn1 in canal epithelium may be context dependent.

We observed a similar expansion of the *Ntn1* expression domain in *Bmp2* conditional mutants to that seen in *Lrig3* nulls, but without the increase in basement membrane breakdown or programmed cell death of the *Lrig3* nulls (Fig. 10B). Although we cannot rule out an increased fusion plate formation in the *Bmp2* mutants as a result of *Ntn1* expansion, a late stage canal resorption analysis indicates that the basement membrane remains intact at the canal rim despite the upregulated *Ntn1* expression (Fig. 6). Thus, the negative regulation of Ntn1 function by *Lrig3* and *Bmp2* is mechanistically different. For example, *Dlx5* is downregulated in the rim domain of the *Bmp2* mutants and this is not the case for *Lrig3* nulls (Abraira et al., 2008). Furthermore, *Nor-1*, another gene expressed in the resorption domain, is not expanded in the *Bmp2* mutants, suggesting that the rim is not co-opted entirely into the resorption domain. Based on these results, we propose that the absence of canals in *Bmp2* conditional knockouts is partially due to upregulation of *Ntn1* function, which affects the canal rim identity (expression of *Dlx5* and *Lmo4* as well as cell proliferation) but does not involve expedited basement membrane breakdown at the rim.

Although there is no evidence of Ntn1 inhibiting Lrig3 (Abraira et al., 2008), *Ntn1* negatively regulates Bmp2 functions, at least in the formation of the anterior canal (Fig. 7). The cellular mechanism for this regulation is not clear. Even though we did not observe an obvious expansion of rim domain identity or upregulated *Bmp2* expression in the *Ntn1* knockout, as postulated based on the genetic results (Figs 7 and 9), it is quite possible that Bmp2 is mediating its effect indirectly via reciprocal inhibition between canal rim and resorption domains (Fig. 10A). Under this scenario, Bmp2 functions in the canal rim. By removing one allele of *Bmp2* in the *Ntn1* knockouts, it reduces the inhibitory effect of the canal rim on the resorption domain and alleviates the non-resorption phenotype in *Ntn1* knockouts (Figs 7 and 10A, Table 2). Such reciprocal inhibition has been described for Lmx1a and Lmo4 recently (Huang et al., 2018). Although Lmx1a and Lmo4 regulate each other in forming transcriptional complexes, the mechanisms underlying the reciprocal inhibition between Bmp2 and Ntn1 will require further investigation. Nevertheless, the combined results in mouse, chicken and zebrafish suggest that Bmp2 has a conserved role in the formation of all three semicircular canals.

## MATERIALS AND METHODS

### Mice and genotyping

The *Bmp2^tm1.1Mis/tm1.1Mis^* (*Bmp2^lox/lox^*) and *Bmp2^+/tm1Brd^* (*Bmp2*^+/−^) mice were maintained on a 129SvEv; C57BL6/J mixed background as previously described (Singh et al., 2008; Zhang and Bradley, 1996). *Bmp2* conditional mutants were generated by mating *Foxg1^cre/+^; Bmp2^+/−^* with *Bmp2^lox/lox^* mice. The *Ntn1^+/Gt(ST629)Byg^* (*Ntn1^+/−^*) mice (Serafini et al., 1996) on C57BL/6J background were obtained from Dr Lisa Goodrich (Harvard Medical School, Boston, MA, USA) and were used to generate *Foxg1^cre/+^; Bmp2^lox/−^;* *Ntn1^+/−^* or *Ntn1^−/−^; Bmp2^+/−^* mice.

### *In situ* hybridization

For *in situ* hybridization, RNA probes for *Bmp4* (Morsli et al., 1998), *Bmp2* (Hwang et al., 2010), *Ntn1* (Abraira et al., 2008), *Dlx5* (Depew et al., 1999) and *Lmo4* (Deng et al., 2006) were prepared as previously described. All *in situ* hybridization results were repeated at least three times with littermate pairs.

### Immunohistochemistry, TUNEL and BrdU labeling

For immunohistochemistry, specimens were fixed in 4% paraformaldehyde for 1 h for anti-pSmad 1/5/8 or overnight at 4°C for anti-laminin staining. The primary antibodies used were anti-pSmad 1/5/8 antibody (a gift from Edward Laufer, Columbia University, NY, USA; 1:1000) and anti-laminin antibody (Millipore, AB2034, 1:500). Secondary antibodies were either Alexa 488 or 568 (Invitrogen, A21206, A10042, 1:200). Apoptotic cells were detected using the TUNEL (Terminal dUTP Nuclear End Labeling) technique (Millipore). For the detection of cell proliferation, the Amersham cell proliferation kit (GE Healthcare) was used. The 5′-bromo-2′deoxyuridine (BrdU) labeling solution (1 ml of a 10 mM BrdU solution per 100 g body weight) was injected intraperitoneally, 2 hours before harvesting. After cryo-sectioning, slides were post-fixed with 4% paraformaldehyde for 10 min at room temperature and treated with an antigen retrieval solution [12.5 ml formamide, 7.5 ml 20×SSC (pH 4.5), 2.5 ml 10% SDS and 2.5 ml dH_2_O] for 30 min at 65°C. The slides were incubated with anti-BrdU antibody (mouse monoclonal, dilution 1:100) in DNAse-1 solution for 2 h at room temperature and then followed with peroxidase conjugated anti-mouse IgG (dilution 1:200) for 1 h. The stable DAB (diaminobenzidine, Invitrogen) solution was applied for detection and methyl green (Vector) was used for counter-staining. BrdU labeled cells were also detected by using a fluorescent-tagged secondary antibody (Invitrogen).

### Data quantification and statistics

To quantify BrdU-positive cells, three representative sections were selected from the vertical canal pouch of E11.75 or anterior and posterior canals of E12.5 ears from wild type (*n*=3 for each age) and mutants (*n*=3 for each age). The average number of BrdU-positive cells per entire epithelial area were calculated. The epithelial areas were measured using ImageJ software (available at https://imagej.nih.gov/ij/index.html). As controls, the average number of BrdU-positive cells per endolymphatic duct epithelial area were also measured and calculated. For statistical analysis, BrdU cell counts were analyzed using Student's *t*-test, and categorized paint fill data were analyzed using Chi-square tests with Yates’ correction.

### qRT-PCR analyses

The dorsal halves of E11.5 to E11.75 *Bmp2* conditional mutant and littermate inner ears (including the vertical canal pouch, endolymphatic duct and some surrounding mesenchyme) were dissected in ice-cold 1× PBS and flash frozen in a slurry of dry ice and methyl butane. RNA was extracted from the frozen tissue using the RNAqueous Micro RNA Isolation Kit (Ambion). The quality and concentration of the RNA samples were evaluated using an Agilent 2100 bioanalyzer and RNA 6000 Nano Kit. RNA of high quality (RIN>8.0) was converted to cDNA with the Superscript III First-Strand cDNA Synthesis system (Life Technologies) using oligo-dT primers. Relative gene expression levels in *Bmp2* conditional mutants and heterozygous control otocysts were evaluated via qRT-PCR performed using Taqman Fast Advanced Master Mix and Taqman Gene Expression Assays on an Applied Biosystems ViiA 7 Real Time PCR system. Each reaction was run in triplicate to assess technical repeatability. Expression levels of target genes (*Bmp2*, *Dlx5*, *Nor1*, *Lmo4* and *Ntn1*) using specific primers (Life Technologies) were normalized to expression levels of reference genes *Gapdh* and *Rpl30*.

## Supplementary Material

Supplementary information
