# Supplementary material for: Genetic interactions support an inhibitory relationship between bone morphogenetic protein 2 and netrin 1 during semicircular canal formation
Source: Development. 2019 Feb 15;146(4):dev174748. doi: 10.1242/dev.174748 (PMC6398446; doi:10.1242/dev.174748)
Supplement: Supplementary information [file develop-146-174748-s1.pdf]

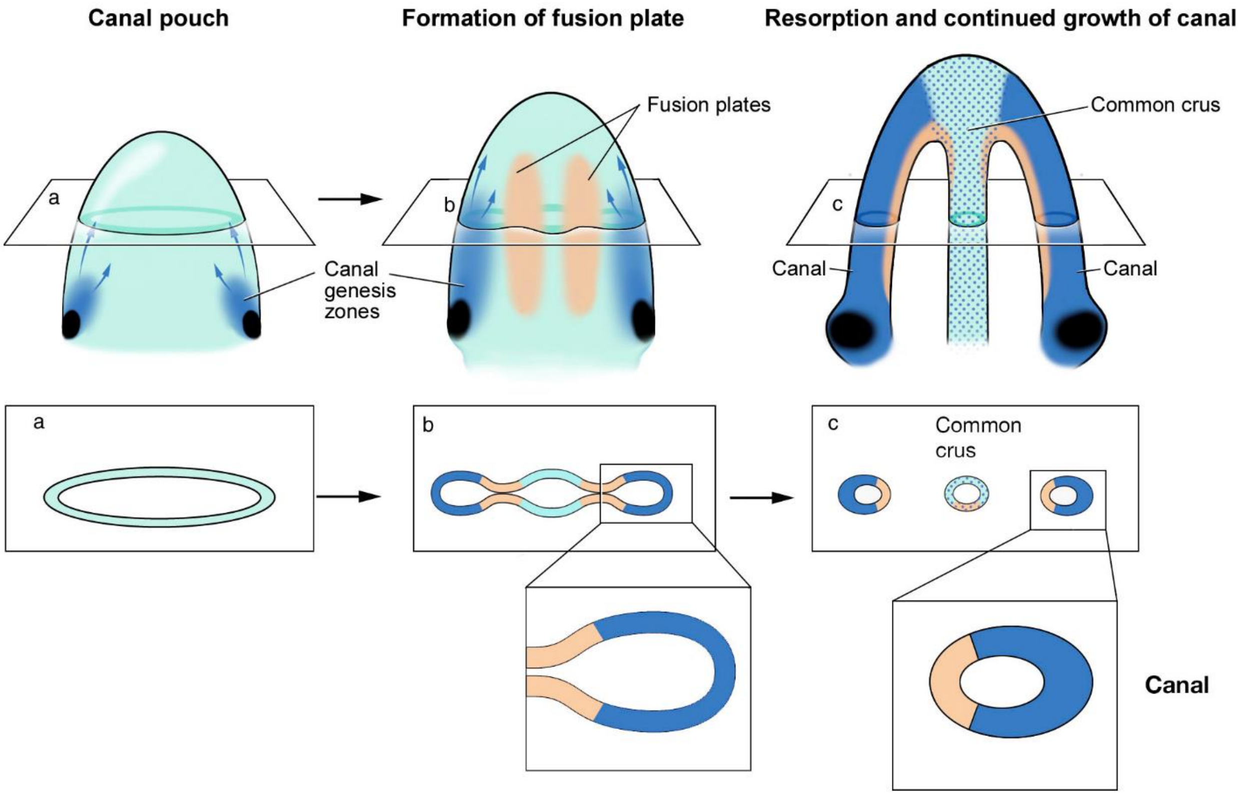

Fig S1. Model of anterior and posterior semicircular canal formation based on studies in chicken.

In the developing canal pouch, a region of epithelium by each presumptive crista (black circle) is induced to form the canal genesis zone (blue color), which gives rise to most of the cells in the two canals and some of the cells in the common crus (dotted blue color). Two regions in the center of the canal pouch (tan color) form the fusion plates in which epithelial cells either disappear or retract, leaving behind the M-shape canals and common crus. Adapted from (Chang et al., 2004; Wu and Kelley, 2012).

Development • Supplementary information

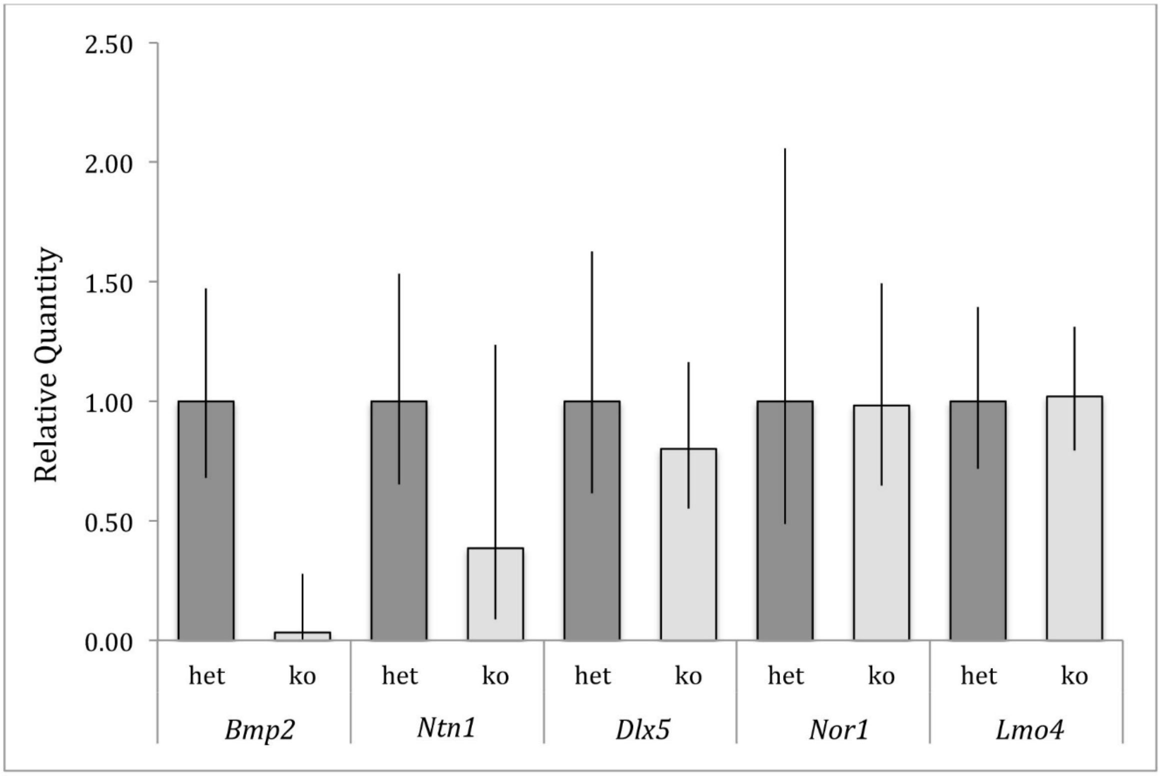

Fig S2. Comparison of *Bmp2* and putative downstream targets of Bmp2 between *Foxg1$^{cre/+}$*; *Bmp2$^{lox/-}$* and *Foxg1$^{cre/+}$*; *Bmp2$^{lox/+}$* vestibules. Relative gene expression levels in canal pouches of *Bmp2* conditional knockout (ko, n=4) and heterozygous controls (n=3) were compared via qPCR. As expected, *Bmp2* ko canal pouches express almost no *Bmp2* compared to controls ($p < 0.05$). However, no statistical differences in expression of *Ntn1*, *Dlx5*, *Nor1*, or *Lmo4* in *Bmp2* conditional knockout versus heterozygote vestibules were detected. Error bars indicate the minimum and maximum RQ values for each group. To quantify the expression level of a particular gene in a sample, the difference between the cycle at which the PCR reaction for that gene reached threshold (C_T) and the average of the C_Ts for *Gapdh* and *Rpl30* for the same sample was defined as the ΔC_T for that sample. Each sample that reproducibly

Development • Supplementary information

failed to reach threshold for a particular target gene was assigned a C_T score of 40 for that gene. For each target gene, mean ΔC_Ts were calculated for ko and control groups and the difference between the ΔC_Ts for the conditional knockout and control groups for a particular target gene was defined as the $\Delta\Delta C_T$ for the ko group. The $\Delta\Delta C_T$ values represent relative expression differences between the two groups and were converted to Relative Quantities (RQ) using the formula $RQ = 2^{-\Delta\Delta CT}$. For each target gene, the RQ value for the ko group was plotted relative to an RQ value of 1 for the control group. The minimum and maximum RQ value in each group are indicated as “error” bars on the graph to demonstrate the range of variability. Differences between the ko and control groups were evaluated using Student’s *t*-tests on the normalized ΔC_T values.

Development • Supplementary information

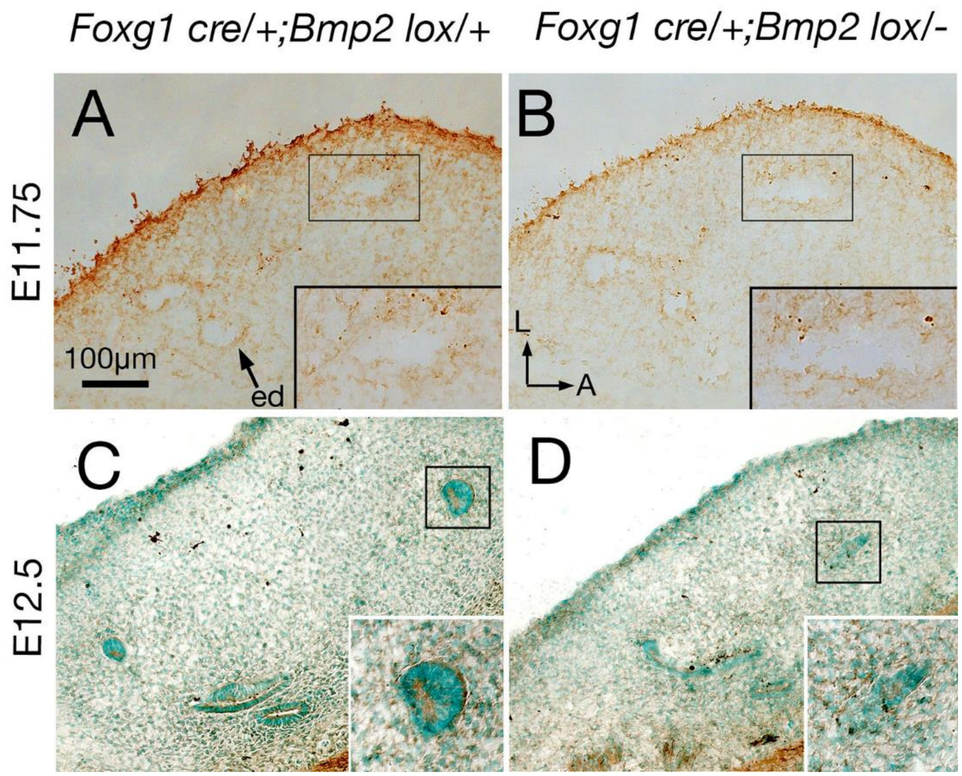

Fig S3. Apoptotic profiles in the canal pouch and canals of *Bmp2* conditional knockout mutants.

Cryostat sections of heterozygous control (A, C) and *Bmp2* conditional knockout (B, D) vestibules at E11.75 (A, B) and E12.5 (C, D). No obvious change in apoptotic profiles or distribution was observed between canal pouches (A, B) and canals (C, D) of controls and mutants. (C) and (D) are counter stained with methyl green after TUNEL detection.

Development • Supplementary information
